# Benefits of Taking Sodium-Glucose Cotransporter 2 Inhibitors in Patients With Type 2 Diabetes Mellitus and Cardiovascular Disease: A Systematic Review

**DOI:** 10.7759/cureus.29069

**Published:** 2022-09-12

**Authors:** Aditi Sarker, Adarsh Srinivas Ramesh, Carlos Munoz, Dawood Jamil, Hadrian Hoang-Vu Tran, Mafaz Mansoor, Samia Rauf Butt, Travis Satnarine, Pranuthi Ratna, Pousette Hamid

**Affiliations:** 1 Family Medicine, California Institute of Behavioral Neurosciences & Psychology, Fairfield, USA; 2 Internal Medicine, California Institute of Behavioral Neurosciences & Psychology, Fairfield, USA; 3 General Medicine, California Institute of Behavioral Neurosciences & Psychology, Fairfield, USA; 4 Pediatrics, California Institute of Behavioral Neurosciences & Psychology, Fairfield, USA; 5 Neurology, California Institute of Behavioral Neurosciences & Psychology, Fairfield, USA

**Keywords:** heart failure., cardiovascular complications, sglt 2 inhibitors, oral hypoglycemic agents, type 2 dm

## Abstract

Type 2 diabetes mellitus (T2DM) is a significant cause of cardiovascular deaths worldwide. There are many oral antihyperglycemic drugs available to treat diabetic patients. Among them, sodium-glucose cotransporter 2 (SGLT2) inhibitors provide effective treatment in all stages of T2DM regardless of blood glucose levels and benefit the cardiovascular system. SGLT2 inhibitors have an additional diuretic effect that reduces blood pressure and hospitalizations and improves heart failure outcomes. This study will assess the efficacy of SGLT2 inhibitors in cardiovascular outcomes in patients with T2DM and cardiovascular disease. Our systematic review followed the Preferred Reporting Items for Systematic Reviews and Meta-Analyses (PRISMA) guidelines and involved a literature search utilizing PubMed and Google Scholar databases. In addition, we thoroughly searched for studies conducted in the last 10 years that corresponded with our outlined inclusion and exclusion criteria. Our search yielded 779 articles. The articles were then quality-checked before inclusion. We ultimately selected six randomized controlled trials and two meta-analyses of research articles after applying the inclusion and exclusion criteria. Our research study included 91,796 T2DM and cardiovascular disease patients. We examined cardiovascular outcomes among these T2DM patients, such as major adverse cardiac events (MACE), blood pressure, heart failure, and hospitalizations. Our study showed that SGLT2 inhibitors significantly reduce weight and blood pressure due to their natriuretic effects. In addition, they also improve heart failure symptoms and reduce hospitalizations.

## Introduction and background

Type 2 diabetes mellitus (T2DM) and cardiovascular disease are the leading causes of increased hospitalization, morbidity, and mortality worldwide [1-3]. In addition, patients with T2DM are at significant risk of developing a progression of renal disease and heart failure [4,5]. Although there is a well-known association between T2DM and heart failure, no known antihyperglycemic agents that can reduce cardiovascular complications in patients with T2DM were described previously. 

Sodium-glucose cotransporter 2 (SGLT2) inhibitors reduce blood glucose levels by inhibiting glucose reabsorption in the proximal tubule. In addition, SGLT2 inhibitors reduce intravascular volume and alter intra-renal hemodynamics by enhancing natriuresis and improving arterial stiffness, which affects blood pressure, body weight, and albuminuria and, therefore, are associated with a reduction in cardiovascular outcomes [6-11]. Explaining the mechanisms by which SGLT2 inhibitors exert their beneficial effects is vital as this knowledge can inform the optimal use of these agents. The U.S. Food and Drug Administration (FDA) has approved several SGLT2 inhibitors for clinical use. Three SGLT2 inhibitors (canagliflozin, empagliflozin, and ertugliflozin) have been studied extensively in cardiovascular outcomes trials [8-10]. In addition, previous studies have found that SGLT2 inhibitors significantly reduced significant adverse cardiovascular events in patients with established atherosclerotic cardiovascular disease (ASCVD) [12]. Therefore, SGLT2 inhibitors are recommended as a second-line agent after metformin by the current North American and European guidelines for patients with ASCVD and heart failure [13,14]. However, the effects of SGLT2 inhibitors on right ventricular structure and function are unknown [15].

SGLT2 inhibitors are not recommended as antihypertensive therapy. However, given that these agents could plausibly affect cardiovascular and renal outcomes positively via direct antihypertensive properties, the specific role of SGLT2 inhibitors in a person with T2DM and hypertension needs to be defined [16]. Therefore, we designed this systematic review to assess the efficacy of SGLT2 inhibitors on cardiovascular outcomes in patients with T2DM and cardiovascular disease. The cardiovascular consequences of interest included hypertension, major adverse cardiovascular events (MACE), congestive heart failure (CHF) hospitalizations, and cardiovascular mortality. Furthermore, by establishing the favorable diuretic profile of SGLT2 inhibitors in managing volume status in heart failure patients, future research can also be considered with respect to SGLT2 inhibitors as an antihypertensive agent among patients without DM.

## Review

Methods

Methodology and Search Strategy

This systematic review was conducted following the Preferred Reporting Items for Systematic Reviews and Meta-Analyses (PRISMA) guidelines [17,18]. We searched the articles on PubMed and Google Scholar databases. We used regular and Medical Subject Headings (MeSH) keywords for the search. The relevant regular keywords were T2DM, oral hypoglycemic agents, SGLT2 inhibitors, cardiovascular complications, and heart failure. We used the following MeSH keywords when exploring PubMed:

("Hypoglycemic Agents/adverse effects"[Majr] OR "Hypoglycemic Agents/classification"[Majr] OR "Hypoglycemic Agents/physiology"[Majr] OR "Hypoglycemic Agents/therapeutic use"[Majr] OR "Hypoglycemic Agents/therapy"[Majr]) OR ("Hypoglycemic Agents/adverse effects"[Mesh:NoExp] OR "Hypoglycemic Agents/classification"[Mesh:NoExp] OR "Hypoglycemic Agents/physiology"[Mesh:NoExp] OR "Hypoglycemic Agents/therapeutic use"[Mesh:NoExp] OR "Hypoglycemic Agents/therapy"[Mesh:NoExp]) AND AND Diabetic complications OR Hyperglycemia OR Macrovascular complications OR Micro vascular complications ("Diabetes Complications/complications"[Majr] OR "Diabetes Complications/diet therapy"[Majr] OR "Diabetes Complications/drug therapy"[Majr] OR "Diabetes Complications/physiopathology"[Majr] OR "Diabetes Complications/prevention and control"[Majr]) OR ("Diabetes Complications/complications"[Mesh:NoExp] OR "Diabetes Complications/diet therapy"[Mesh:NoExp] OR "Diabetes Complications/drug therapy"[Mesh:NoExp] OR "Diabetes Complications/physiopathology"[Mesh:NoExp] OR "Diabetes Complications/prevention and control"[Mesh:NoExp]).

Two investigators (AS and CA) reviewed each article’s title and abstract to determine eligibility independently. Then the analysis by both reviewers was compared and disagreements were resolved by consensus.

Inclusion and Exclusion Criteria

This paper included systematic reviews, meta-analyses, and randomized controlled trials (RCTs), all full-text articles in the English language, involving patients aged >18 years with T2DM and cardiovascular disease irrespective of sex, ethnicity, body mass index, and HbA1c, patients with estimated glomerular filtration rate (eGFR) >30 ml/min/1.73 m^2^, patients with a significant reduction in adverse cardiovascular outcomes (blood pressure, MACE, CHF hospitalizations, cardiovascular mortality) and any changes in right ventricular parameters. We excluded all nonrandomized clinical trials, irrelevant studies that did not include cardiovascular outcomes in diabetic patients, and studies involving a specific ethnicity and sex. The two independent investigators performed data extraction (AS and AR) from the selected studies, which included study design, number of study participants, baseline patient characteristics, use of SGLT2 inhibitors, patients with significant changes in systolic and diastolic blood pressure, and improvement in heart failure and reduction in hospitalization during the study.

Quality Assessment Tools

Two investigators (AS and AR) independently assessed the risk of bias, using the AMSTAR for systematic review and meta-analysis and the Cochrane risk-of-bias tool for RCTs. Table 1 and Table 2 summarize the results of the quality assessment.

**Table 1:** Quality assessment of systematic review and meta-analysis using AMSTAR guidelines AMSTAR: assessment of multiple systematic reviews

Systematic review and meta-analysis	AMSTAR questions checklist	Conclusion
Benham et al. [16]	Yes	High quality
Lo et al. [19]	Yes	High quality

**Table 2:** Quality assessment of RCTs using the Cochrane risk-of-bias tool RCT: randomized controlled trial

RCT	Selection bias	Reporting bias	Performance bias	Detection bias	Attrition bias
Perkovic et al. [20]	Low risk	Low risk	Low risk	Low risk	Low risk
Griffin et al. [21]	Low risk	Low risk	Low risk	Low risk	Low risk
Mordi et al. [22]	Low risk	Low risk	Low risk	Low risk	Low risk
Sarak et al. [23]	Low risk	Low risk	Low risk	Low risk	Low risk
Tikkanen et al. [24]	Low risk	Low risk	Low risk	Low risk	Low risk
Cannon et al. [25]	Low risk	Low risk	Low risk	Low risk	Low risk

Results

Literature Search and Study Selection

**Figure 1:**
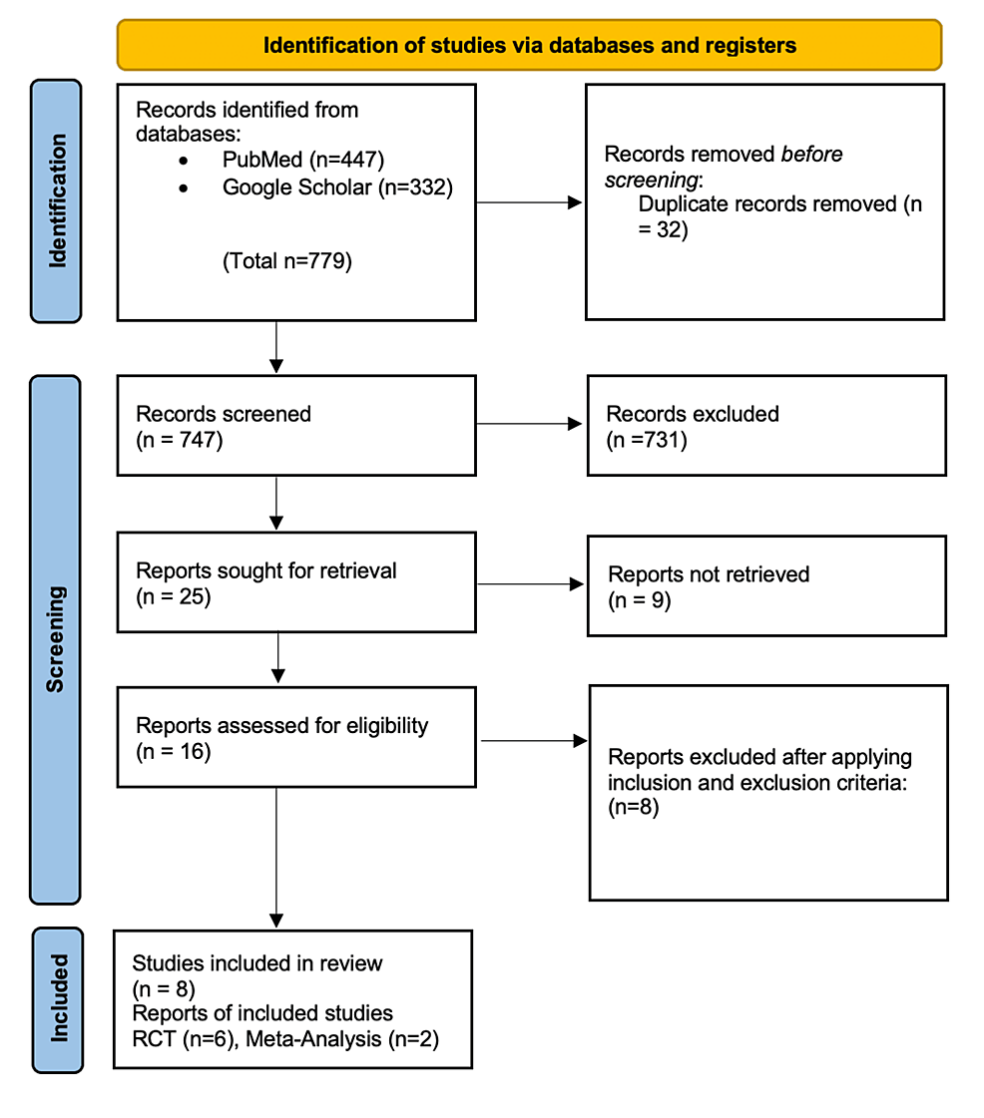
PRISMA flow diagram demonstrating the process of article selection PRISMA: Preferred Reporting Items for Systematic Reviews and Meta-Analysis

Our search strategy results were as follows: we used MeSH keywords on PubMed. For both PubMed and Google Scholar, for filtering, we included studies from 2012-2022 whenever full texts were available; among the article types, we included RCTs, systematic reviews, and meta-analyses. A total of 779 published papers were found by utilizing the initial search criteria. We found 447 articles on PubMed and 332 articles on Google Scholar. We removed a total of 32 duplicate articles before the screening began. After screening the titles for relevance, narrowing down inclusion and exclusion criteria, and duplication removal, we found 16 full-text articles. We identified eight articles as suitable for our research question and eliminated the other eight articles. Figure 1 illustrates the PRISMA 2020 flow chart of article identification and stages of the systematic review.

The included studies' baseline characteristics are shown in Tables 3-7 below. All tables include the name of the author and the year of publication. Out of the eight studies included in our study, six were RCTs, and two studies were systematic reviews and meta-analyses.

**Table 3:** Baseline characteristics of included studies showing author and year of publication, study design, mean age, and patients with type 2 DM DM: diabetes mellitus; RCT: randomized controlled trial; SGLT2: sodium-glucose cotransporter 2

Author and year of publication	Study design	Patient mean age (years): SGLT2 inhibitors	Patient mean age (years): control	Does the study involve patients with type 2 DM?
Benham et al. [16]; 2015	Meta-analysis	52.20–68.50	60	Yes
Lo et al. [19]; 2020	Meta-analysis	62	61	Yes
Perkovic et al. [20]; 2019	RCT	63	62	Yes
Griffin et al. [21]; 2020	RCT	64	63	Yes
Mordi et al. [22]; 2020	RCT	70	69	Yes
Sarak et al. [23]; 2021	RCT	64	64	Yes
Tikkanen et al. [24]; 2019	RCT	61	59	Yes
Cannon et al. [25]; 2020	RCT	65	65	Yes

**Table 4:** Baseline characteristics including author and year of publication, patients with cardiovascular disease, total sample size, sex and ethnicity, number of patients in the control and SGLT2 inhibitor groups, and mean HbA1c in the study population M: male; F: female; SGLT2: sodium-glucose cotransporter 2; HbA1c: hemoglobin A1c

Author and year of publication	Does the study involve patients with cardiovascular disease?	Total sample size, sex, and ethnicity	Number of patients taking SGLT2 inhibitors	Number of patients in the control group	Mean HbA1c in the study population
Benham et al. [16]; 2015	Yes	61,076, irrespective of sex and ethnicity	54,279	6,797	7.17–8.94
Lo et al. [19]; 2020	Yes	17,110, irrespective of sex and ethnicity	10,440	6,670	7.6–8.4
Perkovic et al. [20]; 2019	Yes	4,401, M>F; White>Black>Asian	2,202	2,199	8.4
Griffin et al. [21]; 2020	Yes	20, M>F; Black>White>Asian	14	6	7.5
Mordi et al. [22]; 2020	Yes	23, M>F; White>Black>Asian	12	11	7.9
Sarak et al. [23]; 2021	Yes	97, M>F; White>Black>Asian	49	48	7.9
Tikkanen et al. [24]; 2019	Yes	823, M>F; White>Black>Asian	552	271	7.9
Cannon et al. [25]; 2020	Yes	8246, M>F; White>Black>Asian	5,499	2,747	8.2

**Table 5:** Baseline characteristics including author and year of publication, mean eGFR, mean BMI, and addition of loop diuretics in the study population eGFR: estimated glomerular filtration rate; BMI: body mass index

Author and year of publication	Mean eGFR in the study population, ml/min/1.73 m^2^	Mean BMI in the study population, kg/m^2^	Addition of loop diuretics
Benham et al. [16]; 2015	<60	32.4	Yes
Lo et al. [19]; 2020	<60	34.5	No
Perkovic et al. [20]; 2019	56.3	31.3	Yes
Griffin et al. [21]; 2020	>45	37.2	Yes
Mordi et al. [22]; 2020	65	33.9	Yes
Sarak et al. [23]; 2021	>60	26.7	No
Tikkanen et al. [24]; 2019	84	33	No
Cannon et al. [25]; 2020	76	31.9	No

**Table 6:** Baseline characteristics including author and year of publication, number of patients admitted to the hospital for heart failure and cardiovascular death, the effect of SGLT2 inhibitors on natriuresis, and changes in right ventricular parameters SGLT2: sodium-glucose cotransporter 2

Author and year of publication	Hospitalization for heart failure	Cardiovascular death	Effect of SGLT2 inhibitors on natriuresis	Changes in right ventricular parameters
Benham et al. [16]; 2015	32% reduction in congestive heart failure hospitalizations in patients with SGLT2 inhibitors compared to placebo	18% reduction in cardiovascular death in patients with SGLT2 inhibitors compared to placebo	Yes	_
Lo et al. [19]; 2020	_	_	_	_
Perkovic et al. [20]; 2019	179 in canagliflozin and 253 in the placebo group	110 in canagliflozin and 140 in the placebo group	_	_
Griffin et al. [21]; 2020	_	_	Yes	_
Mordi et al. [22]; 2020	_	_	Yes	_
Sarak et al. [23]; 2021	_	_	_	22.8 in empagliflozin and 20.7 in the placebo
Tikkanen et al. [24]; 2019	_	_	_	_
Cannon et al. [25]; 2020	139/5,499 in the ertugliflozin group compared to 99/2,747 in the placebo group	444 of 5,499 patients (8.1%) in the ertugliflozin group compared to 250 of 2,747 patients (9.1%) in the placebo group	No	_

**Table 7:** Baseline characteristics including author and year of publication, the effect of SGLT2 inhibitors on HTN, body weight and MACE outcome, follow-up, and conclusion SGLT2: sodium-glucose cotransporter 2; HTN: hypertension; MACE: major adverse cardiac events

Author and year of publication	Effect of SGLT2 inhibitors on MACE outcome	Effect of SGLT2 inhibitors on HTN and body weight	Follow-up	Conclusion
Benham et al. [16]; 2015	12% reduction in 3-point MACE compared to placebo	_	12–338 weeks	SGLT2 inhibitors caused a significant reduction in MACE, heart failure hospitalizations, and mortality. They also enhanced natriuresis
Lo et al. [19]; 2020	490/4,687 in empagliflozin and 585/5,795 in canagliflozin compared to 282/2,333 and 426/4,347 in placebo, respectively	_	6–12 weeks	SGLT2 inhibitors caused a significant reduction in MACE
Perkovic et al. [20]; 2019	_	_	2.62 years	Canagliflozin is associated with a lower risk of heart failure hospitalization and cardiovascular mortality than placebo
Griffin et al. [21]; 2020	_	_	14 days	Empagliflozin modestly enhanced natriuresis and has synergistic effects when combined with loop diuretics
Mordi et al. [22]; 2020	_	_	6 weeks	Empagliflozin causes significant weight loss
Sarak et al. [23]; 2021	_	_	6 months	Empagliflozin has no impact on right ventricular myocardial impaction
Tikkanen et al. [24]; 2019	_	Yes	12 weeks	Empagliflozin caused a significant reduction in 24-hour blood pressure compared to a placebo
Cannon et al. [25]; 2020	_	_	3.5 years	Ertugliflozin caused a significant reduction in MACE, heart failure hospitalizations, and mortality

Discussion

This systematic review of six RCTs and two meta-analyses explored the relationship between T2DM and the magnitude reduction of cardiovascular outcomes, such as cardiovascular mortality, MACE, and CHF hospitalizations of SGLT2 inhibitors compared to placebo, in a population with eGFR >30 ml/min/1.73 m^2^ with or without the use of diuretics, average age >60 years, irrespective of sex and ethnicity, range of HbA1c 7-8.9%, and BMI between 26-37 kg/m^2^. Our study showed that SGLT2 caused a significant increase in diuresis/natriuresis. SGLT2 inhibitors also caused considerable weight loss [26,27]. However, the interplay between T2DM and heart failure is complex and multifactorial, and mechanisms of potential benefit in heart failure may extend beyond simple intravascular volume loss [27]. There is a linear relationship between these cardiovascular outcomes and the difference in change in systolic and diastolic blood pressure, improving cardiorenal hemodynamics and potentially leading to better heart failure outcomes.

With their diuretic-like effect, SGLT2 inhibitors exhibit a similar response to thiazide and loop diuretics, which reduce blood pressure through natriuresis, leading to decreased plasma volume [19-25,28-30]. Hypertension is a common comorbidity in patients with T2DM and increases the risk of cardiovascular complications [31]. Therefore, further investigations are needed to explore whether they can be considered for managing hypertension in patients with or without type 2 diabetes. Below is a discussion of published research articles that compare the analysis of different authors, which can give us greater insights into the efficacy of SGLT2 inhibitors in cardiovascular outcomes.

Benham et al. conducted a systematic review and meta-analysis of 40 RCTs to assess cardiovascular outcomes, such as cardiovascular mortality, 3-point MACE (a composite of cardiovascular death, stroke, and myocardial infarction), and CHF hospitalizations [16]. In this study, the authors revealed that a total of 551 deaths from cardiovascular causes were reported among the 25,458 participants treated with an SGLT2 inhibitor compared to 536 events among 18,719 controls. The authors also reported that treatment with an SGLT2 inhibitor compared to the placebo led to a 19% reduction in cardiovascular mortality, a 12% reduction in 3-point MACE, and a 32% reduction in CHF hospitalizations. This study also assessed the diuretic effect of SGLT2 inhibitors, which reduce blood pressure through natriuresis and decrease plasma volume. When SGLT2 inhibitors were combined with thiazide or loop diuretics, no additive blood pressure reduction was observed. However, additive blood pressure reduction was observed when SGLT2 inhibitors were combined with renin-angiotensin-aldosterone system inhibitors, beta-blockers, and calcium channel blockers.

Lo et al. conducted a systematic review and meta-analysis to assess the efficacy of SGLT2 inhibitors in cardiovascular and renal outcomes, especially with eGFR <60 ml/min/1.73 m^2^ in T2DM patients. This study observed a significant reduction in MACE using SGLT2 inhibitors compared to a placebo [19]. However, the study did not assess the rate of hospitalizations from hypertensive heart failure and mortality rate.

Perkovic et al. conducted an RCT and showed various advantages of using SGLT2 inhibitors, especially canagliflozin, among T2DM patients with cardiovascular and chronic kidney disease [20]. The study showed a significant decrease in renal and cardiovascular death in the canagliflozin group than placebo (43.2 and 61.2 per 1,000 patient-years, respectively), which resulted in a 30% lower relative risk. In addition, patients in the canagliflozin group had a lower risk of hospitalization for heart failure and myocardial infarction. However, this study did not analyze the natriuretic effect of canagliflozin.

However, Griffin et al. conducted an RCT study of the effect of empagliflozin on T2DM and stable heart failure patients [21]. They showed significantly enhanced natriuresis and a synergistic natriuretic effect when combined with loop diuretics. Furthermore, empagliflozin was not associated with worsening potassium wasting, unlike traditional diuretics. In addition, the natriuretics reduced blood and plasma volume and were not associated with a decline in glomerular filtration rate. However, the study did not provide much information on the hospitalization rate for heart failure and cardiovascular death among T2DM patients.

Mordi et al. used empagliflozin in combination with loop diuretics to assess the renal and cardiovascular effects on T2DM and heart failure patients [22]. Their RCT study observed that empagliflozin caused a significant increase in 24-hour urine volume (an electrolyte-free water clearance) without significant natriuresis. Empagliflozin also caused weight loss, no significant renal impairment, and electrolyte disturbance. Unfortunately, the study had a short follow-up and a small sample size. However, the study included patients with T2DM and chronic heart failure with ejection fraction <50%, and the study did not assess these patients' hospitalization rate and cardiovascular death.

Sarak et al. conducted a study to assess the effects of empagliflozin on right ventricular parameters and functions by using cardiac MRI (cMRI) among T2DM patients with previous myocardial infarction [23]. cMRI provides a standard assessment of right ventricular structure and function. T2DM affects right ventricular remodeling and systolic and diastolic function, even in preserved left ventricular ejection fraction (LVEF) [32]. The study did not show any impact of empagliflozin on right ventricular myocardial infarction (RVMI) and right ventricular volumes in T2DM and coronary artery disease patients. In addition, this study did not assess the effects of empagliflozin on natriuresis, hospitalization rate in decompensated heart failure patients, and cardiovascular death.

Tikkanen et al. conducted an RCT study using empagliflozin in T2DM patients with hypertension [24]. The authors observed that empagliflozin caused a significant reduction in 24-hour (daytime and nighttime) both systolic and diastolic blood pressure compared to placebo. In addition, the authors also assessed that empagliflozin is also associated with a significant reduction in body weight and HbA1c. The follow-up period of the study was 12 weeks. However, the study did not assess empagliflozin's effect on diuresis, heart failure patients' hospitalization rate, and cardiovascular death.

Cannon et al. conducted an RCT with ertugliflozin in T2DM patients and established ASCVD [25]. This study assessed MACE (the primary outcome) that occurred in 653 out of 5,493 patients (11.9%) in the ertugliflozin group compared to 327 of 2,745 patients (11.9%) in the placebo group. The study also assessed deaths from hospitalizations for heart failure that occurred in 444 of 5,499 patients (8.1%) in the ertugliflozin group and compared to 250 of 2,747 patients (9.1%) in the placebo group. However, this study was non-inferior to placebo concerning MACE. In addition, the trial groups did not significantly differ in terms of death from cardiovascular causes or heart failure.

The prevalence of patients with a preserved ejection fraction is increasing and associated with impairment of function similar to those with heart failure and reduced ejection fraction patients. Although SGLT2 inhibitors were initially developed to treat T2DM patients, they provided remarkable benefits among heart failure patients, including preserved and reduced ejection fraction [33]. We would also like to mention two clinical trials published in the New England Journal of Medicine (NEJM): The EMPEROR-Preserved trial (Empagliflozin Outcome Trial in Patients with Chronic Heart Failure with Preserved Ejection Fraction) and the DELIVER trial (Dapagliflozin Evaluation to Improve the Lives of Patients with Preserved Ejection Fraction Heart Failure). In the EMPEROR-Preserved trial, it was demonstrated that empagliflozin reduced the risk of cardiovascular death or heart failure hospitalizations in both patients with a mildly reduced ejection fraction (LVEF of more than 40%) and a preserved ejection fraction (LVEF of more than 50%) [34]. In the DELIVER trail, dapagliflozin was compared with a placebo in patients with heart failure and a mildly reduced or preserved ejection fraction. It showed that dapagliflozin resulted in a lower risk of primary composite outcome or cardiovascular death than placebo [35]. However, it is essential to identify the similarities and distinctions as the design of the DELIVER trail was similar to that of the EMPEROR-Preserved trail. Both trials tested a selective oral SGLT2 inhibitor in their analysis of patients with or without diabetes, heart failure, and an LVEF of more than 40%, with similar inclusion and exclusion criteria and a similar primary composite outcome. Here, the editorial highlighted the main feature of DELIVER trial that distinguished it from the EMPEROR-Preserved trial was the inclusion of patients who previously had an LVEF of 40% or less that subsequently improved to more than 40% after diuresis sufficiently, called patients with heart failure and improved LVEF [33]. Although such patients are typically excluded from clinical trials of treatments for heart failure and a preserved ejection fraction, recent reviews have stated that patients with heart failure and improved LVEF have worse outcomes than patients with heart failure, and LVEF has improved to within the normal range, or even patients without a history of heart failure. It also showed that effects worsen when disease-modifying therapy is discontinued in patients with heart failure and an improved LVEF. The DELIVER trial showed that adding an SGLT2 inhibitor provides further benefit [33].

We believe that this systematic review has notable strengths. In this study, we included a large population. We explored the potential relationship between the cardioprotective effects of SGLT2 inhibitors and the magnitude of blood pressure reduction. However, our study has some limitations as well, primarily due to some of the exclusion criteria, such as accepting only studies in the English language, including only those studies conducted in the last 10 years and those with a population with eGFR >30 ml/min/1.73 m^2^, variation in the follow-up of patients in different studies, and genital mycotic infections occurring more frequently among women and men in either SGLT2 inhibitors dose group than among those in the placebo group.

## Conclusions

Our findings confirm that SGLT2 inhibitors are associated with a significant reduction in MACE, heart failure-related hospitalizations, and cardiovascular mortality in patients with T2DM. They do not have any impact on RVMI. Furthermore, natriuresis is likely a major contributing factor in the observed improvements in cardiovascular outcomes. Therefore, this ideal diuretic profile may offer significant advantages in managing volume status in patients with hypertensive heart failure. Hence, we recommend that future studies be conducted on the efficacy of SGLT2 inhibitors as an antihypertensive agent in controlling hypertension in patients without diabetes.
